# Stroke incidence increases with diabetic retinopathy severity and macular edema in type 1 diabetes

**DOI:** 10.1186/s12933-024-02235-w

**Published:** 2024-04-25

**Authors:** Marika I Eriksson, Kustaa Hietala, Paula Summanen, Valma Harjutsalo, Jukka Putaala, Anni Ylinen, Stefanie Hägg-Holmberg, Per-Henrik Groop, Lena M Thorn, on behalf of the FinnDiane Study

**Affiliations:** 1 Folkhälsan Institute of Genetics, Folkhälsan Research Center, Helsinki, Finland; 2 Department of Nephrology, University of Helsinki and Helsinki University Hospital, Biomedicum Helsinki Haartmaninkatu 8, Helsinki, FIN-00290 Finland; 3 Research Program in Clinical and Molecular Metabolism, University of Helsinki, Helsinki, Finland; 4 Hospital Nova of Central Finland, Jyväskylä, Finland; 5 Department of Ophthalmology, University of Helsinki and Helsinki University Hospital, Helsinki, Finland; 6 Department of Neurology, University of Helsinki and Helsinki University Hospital, Helsinki, Finland; 7 Department of Diabetes, Central Clinical School, Monash University, Melbourne, VIC Australia; 8 Department of General Practice and Primary Health Care, University of Helsinki and Helsinki University Hospital, Helsinki, Finland

**Keywords:** Type 1 diabetes, Stroke, Cerebrovascular complications, Diabetic retinopathy, Macular edema

## Abstract

**Background:**

As the retina is suggested to mirror the brain, we hypothesized that diabetic retinopathy and macular edema are indicative of stroke risk in type 1 diabetes and sought to assess this association in individuals with type 1 diabetes.

**Methods:**

We included 1,268 adult FinnDiane Study participants with type 1 diabetes (age 38.7 ± 11.8 years, 51.7% men vs. 48.3% women, and 31.5% had diabetic kidney disease), data on baseline diabetic retinopathy severity, and first stroke during our observational follow-up. Retinopathy was graded by the Early Treatment Diabetic Retinopathy Study (ETDRS) scale, and macular edema as clinically significant (CSME) or not. Strokes identified from registries were confirmed from medical files. Adjusted hazard ratios (HR) for stroke by retinopathy severity and CSME were calculated by Cox models adjusted for clinical confounders, including diabetic kidney disease.

**Results:**

During median 18.0 (14.1–19.3) follow-up years, 130 strokes (96 ischemic, 34 hemorrhagic) occurred. With no–very mild (ETDRS 10–20) retinopathy as reference, the adjusted HR for stroke was 1.79 (95%CI 1.02–3.15) in non-proliferative (ETDRS 35–53), and 1.69 (1.02–2.82) in proliferative (ETDRS 61–85) retinopathy. Corresponding adjusted HR for ischemic stroke was 1.68 (0.91–3.10) in non-proliferative and 1.35 (0.77–2.36) in proliferative retinopathy. The adjusted HR for hemorrhagic stroke was 2.84 (0.66–12.28) in non-proliferative and 4.31 (1.16–16.10) in proliferative retinopathy. CSME did not increase HR for any stroke type after adjustment for clinical confounders (data not shown).

**Conclusions:**

Stroke incidence increases with the severity of diabetic retinopathy independently of comorbid conditions, including diabetic kidney disease.

**Supplementary Information:**

The online version contains supplementary material available at 10.1186/s12933-024-02235-w.

## Background

Type 1 diabetes is associated with a three- to sixfold risk of stroke [1–3]. In the general population, including individuals with diabetes, an array of retinal vascular abnormalities have been associated with increased stroke risk [4]. Less is known about this association in type 1 diabetes, although it has been suggested that the retinal vessels mirror those of the brain in type 1 diabetes as well [4, 5]. Diabetic retinopathy is, thus, of interest when assessing risk factors for stroke.

In the Finnish Diabetic Nephropathy (FinnDiane) Study, we have shown that in people with type 1 diabetes, the stroke risk, particularly regarding hemorrhagic stroke, is increased in those with severe diabetic retinopathy, even after adjustment for the presence of diabetic kidney disease [6]. Yet, regarding other common retinal complications, such as non-proliferative diabetic retinopathy and macular edema, their role in stroke is not known in type 1 diabetes. The association between diabetic retinopathy severity and incident stroke has previously been studied in type 2 diabetes [7]. In these individuals, any diabetic retinopathy, ranging from mild non-proliferative to proliferative disease, has been linked to an increased risk of ischemic or hemorrhagic stroke when compared to peers with no corresponding diabetic retinopathy. In the same study, stroke risk was further increased with the increase in retinopathy severity [7]. 

Furthermore, in a meta-analysis examining cardiovascular disease in individuals with type 2 diabetes, diabetic retinopathy, as well as macular edema, were identified as risk factors for adverse cardiovascular outcomes, including stroke. The number of strokes within the pooled cohort, however, was not sufficient for a separate analysis of strokes or stroke subtypes [8]. 

The association between stroke and diabetic eye disease might, however, differ in type 1 compared to type 2 diabetes. Stroke in type 1 diabetes is more often of microvascular etiology and related to so-called cerebral small-vessel disease [9]. In the general population, cerebral microbleeds, which are a manifestation of small-vessel disease, have been found to predict both ischemic and hemorrhagic stroke. Moreover, the risk seems to increase with the number of microbleeds [10]. Whether this translates to type 1 diabetes is unknown, but as cerebral small-vessel disease is observed in one-third of middle-aged adults with type 1 diabetes [11], it is of interest to consider different etiologies, such as small vessel disease, when assessing risk factors for stroke in type 1 diabetes.

In type 1 diabetes, cerebral microbleeds have been associated with proliferative diabetic retinopathy [5] and, additionally, the number of cerebral microbleeds increases with retinopathy severity [12]. A question of interest is whether this association might stretch beyond asymptomatic microbleeds, i.e., cerebral small-vessel disease. The severity of diabetic eye disease could be indicative of stroke risk and/or individuals especially prone to a certain stroke subtype, e.g., stroke of small-vessel origin. To test this hypothesis, our objective was to examine the link between diabetic retinopathy severity and stroke of different subtypes in participants with type 1 diabetes. Furthermore, we sought to explore if macular edema is associated with stroke.

## Methods

### Participants

All participants are part of the ongoing FinnDiane Study, which is a Finnish nationwide multicenter study, aiming to uncover risk factors for diabetic complications of type 1 diabetes. By the end of 2017, this observational follow-up study consisted of roughly 5,000 adults with type 1 diabetes, defined as age < 40 years at diabetes onset and insulin initiated within one year from diagnosis. The design has been described in detail previously [13].

**Fig. 1:**
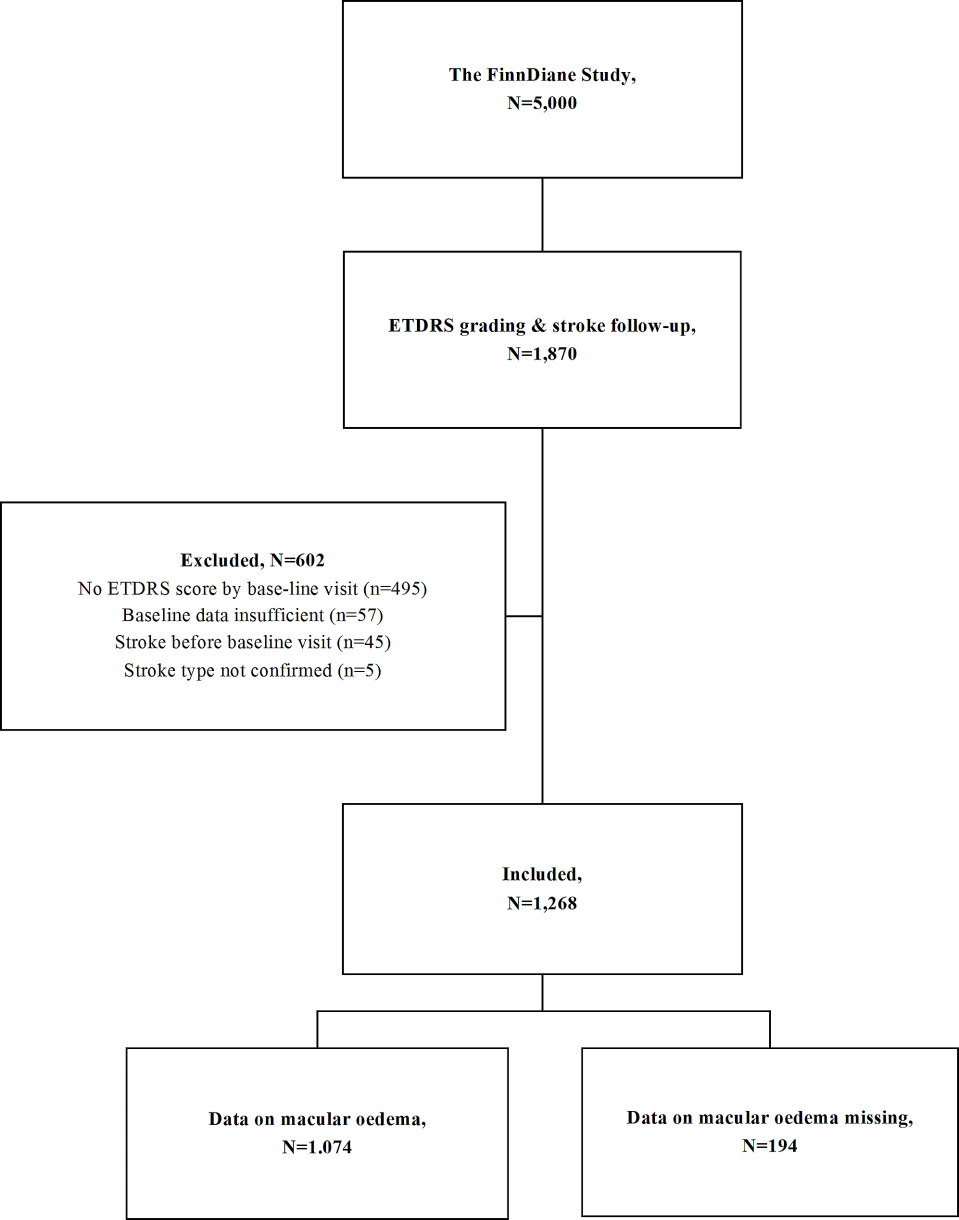
Flow chart of participant selection The flow chart illustrates the selection process for the 1,268 participants included in the study. All participants are part of the Finnish Diabetic Nephropathy (FinnDiane) Study, which by 31.12.2017 included 5,000 participants with type 1 diabetes.

Comprehensive information about diabetic retinopathy was collected for a subset of participants (*n* = 1,983) entering the FinnDiane study before January 2012, as described in one of our previous reports [14]. Of these, we excluded participants lacking information on retinopathy severity by the time of the baseline visit (*n* = 495), participants with insufficient clinical baseline data (*n* = 57), history of stroke (*n* = 45), and those with insufficient information on stroke or stroke type (*n* = 5) during follow-up (Fig. 1). This rendered us a cohort of 1,268 participants with type 1 diabetes, eligible for this study.

Compared to the rest of the FinnDiane Study cohort (*n* = 3,687), participants included in this study did not differ regarding age or sex. Diabetic kidney disease was more prevalent among those included. Supplementary Table 1 presents baseline characteristics and incident strokes for the included vs. excluded participants. (see Supplementary Table 1)

### Clinical characteristics and outcome

Baseline study visits were conducted by the end of 2006. Visits included a thorough clinical examination, assessment of medical history, diabetic complication status, current medication, and questionnaires concerning lifestyle, including smoking. Participants were categorized as either men or women. Smoking was defined as regular smoking of at least one cigarette per day. Based on the questionnaires participants were categorized as current smokers, having a history of smoking, or no history of smoking. In addition, the study visit included anthropometrics and blood pressure measurements. Blood samples were drawn for the analysis of creatinine, HbA1c, lipids, and lipoproteins. Urine was collected for assessment of urinary albumin excretion. We defined diabetic kidney disease as having an albumin excretion rate ≥ 200 μg/min in an overnight urine collection or ≥ 300 mg/24 h in 24-hour collections, in two out of three urine collections. Kidney replacement therapy was also considered diabetic kidney disease. Coronary artery disease at baseline was defined as a history of acute myocardial infarction or coronary revascularization. Peripheral arterial disease was defined as a history of peripheral revascularization or amputation.

Diabetic retinopathy severity was graded on the Early Treatment Diabetic Retinopathy Study (ETDRS) scale [15] by an ophthalmologist (K.H.), based on the participants’ fundus images and medical records, and has been described in our previous work [14]. For the current study, we used retrospective data collections, including only ETDRS scoring before or at baseline to ascertain the minimum severity grade possible at baseline. When scores from several time points were available, we chose the score given closest to or at baseline for analysis. Participants were assigned into three groups based on the ETDRS score. An ETDRS score < 35, was categorized as “no–very mild retinopathy”. Mild to severe non-proliferative diabetic retinopathy, indicated by ETDRS ≥ 35 and < 61, was categorized as “non-proliferative retinopathy”, while proliferative diabetic retinopathy, indicated by ETDRS ≥ 61, was categorized as “PDR” [15]. .

Data on macular status at baseline were available for 1,074 (84.7%) of the included participants. Clinically significant macular edema (CSME) was defined following the ETDRS classification protocol [16] and the dataset has been described in detail in our previous work [17].

Cerebrovascular events during follow-up were identified from the national hospital discharge register (National Care Register of Health Care, Finnish Institute for Health and Welfare) until 31 December 2017. Events registered as stroke (ICD-10: I60–I64; ICD-8, ICD-9: 430–434) were further verified from the participants’ medical records from the discharging hospital, and strokes were classified according to etiology: ischemic stroke or hemorrhagic stroke. Ischemic stroke was further subdivided into microvascular (lacunar stroke) and macrovascular (non-lacunar stroke), or as undefined ischemic stroke when distinction was not possible. Hemorrhagic strokes were subtyped as intracerebral or subarachnoid hemorrhages. Data on deaths during the follow-up time were obtained from the Finnish Cause of Death Register, Statistics Finland. Follow-up time was calculated from the baseline study visit until the date of first stroke, death, or end of follow-up on 31 December 2017.

### Statistical analysis

All analyses were performed using R version 4.2.3 (2023-03-15 ucrt) [18]. For the comparison of baseline characteristics in different groups, we used parametric tests (one-way ANOVA, t-test for two sample analysis) when data were normally distributed and otherwise non-parametric tests (Kruskal-Wallis test, Wilcoxon test for two sample analysis). We analyzed categorical data with the chi-squared test or Fisher’s exact test if observations were ≤ 5 in one of the groups. Observations with missing values were omitted from the analysis.

We calculated the incidence rate as the number of events per 100,000 person-years with a 95% confidence interval (CI) using a Poisson distribution. Cumulative events were plotted with the Kaplan-Meier method and log-rank tested using the R package survminer [19]. All outcomes of interest were analyzed separately by Cox regression models. Potential prognostic variables which were significant in univariate analysis of stroke, were entered as independent variables in the Cox models. When several variables represented the same characteristic and/or there was multicollinearity between variables, only one of the corresponding variables was included in the adjusted analysis. Age at onset of diabetes was included based on assumed clinical relevance, regardless of significance in the parametric test. Variables with missing values, e.g., smoking status, were excluded from the analysis. Cox regression and testing of the assumption of proportional hazards were performed with the survival package [20, 21]. Competing risk analysis including tests for comparing the cumulative incidence for competing risks, and Fine-Gray regression modeling of subdistribution functions in competing risk, were performed with the R package cmprsk [22]. The subdistribution hazard was analyzed in a Fine-Gray model with the same covariates as in the Cox regression models.

## Results

### Clinical characteristics at baseline

The participants’ mean age was 38.7 ± 11.8 years, duration of diabetes 25.5 ± 9.7 years, and HbA1c 70.7 ± 16.1 mmol/mol (8.6 ± 1.5%). Of the participants, 1,119 (88.2%) had an ETDRS score of 20 or higher, indicating any diabetic retinopathy. Table 1 presents baseline characteristics for the participants grouped by retinopathy severity.

**Table 1:** Clinical characteristics presented by diabetic retinopathy severity at baseline

	*n* in analysis	No–very mild diabetic retinopathy	Non-proliferative diabetic retinopathy	Proliferative diabetic retinopathy	*P*
**Baseline data**					
n		575	203	490	
Women	1,268	312 (54.3)	77 (37.9)	223 (45.5)	< 0.001
Age, years	1,268	33.7 [26.1, 43.4]	39.6 [32.6, 47.7]	41.7 [34.4, 48.8]	< 0.001
Duration of diabetes, years	1,268	21.1 ± 9.3	25.8 ± 8.1	30.5 ± 8.1	< 0.001
Age at onset of diabetes, years	1,268	12.9 [7.9, 19.1]	12.8 [8.7, 19.7]	10.2 [6.6, 15.2]	< 0.001
Body mass index, m^2^/kg	1,266	25.1 ± 3.2	25.5 ± 4.0	25.7 ± 4.0	0.052
HbA_1C_, %(mmol/mol)	1,268	8.6 ± 1.5 (70 ± 16)	8.7 ± 1.5 (71 ± 16)	8.6 ± 1.5 (71 ± 16)	0.672
Total cholesterol, mmol/l	1,268	4.9 ± 1.0	5.2 ± 1.0	5.3 ± 1.0	< 0.001
LDL cholesterol, mmol/l	1,268	3.1 ± 0.9	3.3 ± 0.8	3.4 ± 0.9	< 0.001
HDL cholesterol, mmol/l	1,268	1.3 ± 0.4	1.3 ± 0.4	1.2 ± 0.4	< 0.001
Triglycerides, mmol/l	1,268	1.0 [0.8, 1.5]	1.1 [0.8, 1.6]	1.3 [0.9, 1.9]	< 0.001
Use of lipid lowering medication, n (%)	1,267	32 (5.6)	24 (11.8)	91 (18.6)	< 0.001
Systolic blood pressure, mmHg	1,268	130 ± 16	137 ± 19	143 ± 21	< 0.001
Diastolic blood pressure, mmHg	1,265	80 [72, 85]	81 [76, 88]	82 [76, 90]	< 0.001
Use of antihypertensive medication, n (%)	1,267	146 (25.4)	123 (60.6)	392 (80.2)	< 0.001
Diabetic kidney disease, n (%)	1,268	65 (11.3)	61 (30.0)	319 (65.1)	< 0.001
Coronary artery disease, n (%)	1,268	13 (2.3)	11 (5.4)	56 (11.4)	< 0.001
Peripheral arterial disease, n (%)	1,268	8 (1.4)	8 (3.9)	59 (12.0)	< 0.001
Smoking status, n (%)	1,246				< 0.001
Current smoker		141 (24.9)	50 (25.1)	112 (23.3)	
History of smoking		109 (19.3)	53 (26.6)	149 (31.0)	
No history of smoking		316 (55.8)	96 (48.2)	220 (45.7)	
**Follow-up data**					
Any stroke, n (%)	1,268	26 (4.5)	25 (12.3)	79 (16.1)	< 0.001
Ischemic stroke	1,268	23 (4.0)	20 (9.8)	53 (10.8)	< 0.001
Type of ischemic stroke	96				0.492
Lacunar stroke		8 (34.8)	10 (50.0)	24 (45.3)	
Non-lacunar stroke		5 (21.7)	6 (30.0)	16 (30.2)	
Undefined ischemic stroke		10 (43.5)	4 (20.0)	13 (24.5)	
Hemorrhagic stroke, n (%)	1,268	3 (0.5)	5 (2.5)	26 (5.3)	< 0.001
Type of hemorrhagic stroke	34				0.146
Intra cerebral hemorrhage		1 (33.3)	4 (80.0)	22 (84.6)	
Sub-arachnoid hemorrhage		2 (66.7)	1 (20.0)	4 (15.4)	
Death during follow-up, n (%)	1,268	43 (7.5)	34 (16.7)	167 (34.1)	< 0.001

Data are presented as mean ± standard deviation, median [quartiles], and n (%). Normally distributed continuous variables have been tested with one way ANOVA, non-normal variables with Kruskal-Wallis test. Categorical data were analyzed with the chi-squared test or Fisher’s exact test if observations were ≤ 5 in one of the groups.

When comparing the groups, age, duration of diabetes, systolic and diastolic blood pressure, prevalence of diabetic kidney disease, and kidney replacement therapy were increased with increasing severity of diabetic retinopathy. Also, the lipid profile differed between the groups and the use of lipid-lowering medication was most prevalent in the PDR group (ETDRS ≥ 61). (Table 1)

Of the 1,074 participants with available information on macular status, 534 (50.6%) had some degree of macular edema at baseline, and 386 (35.9%) had CSME. The largest proportion of CSME, 48.9% (*n* = 261), was observed in participants with PDR. Of the rest, 15.0% (*n* = 80) had CSME and non-proliferative retinopathy, and 8.4% (*n* = 45) no–very mild retinopathy. Compared to participants with available macular data, those without data were younger, a larger proportion were women, and they had a lower prevalence of diabetic kidney disease. Clinical characteristics by the status of macular edema are presented in more detail in an additional file. (see Supplementary Table 2).

Participants with CSME were older than those without CSME and had a longer duration of diabetes. The use of lipid-lowering medication was more prevalent in those with CSME, they had higher LDL cholesterol, higher systolic blood pressure, more frequent use of antihypertensive medication, and a more often had diabetic kidney disease. (Supplementary Table 2)

### Endpoints during follow-up

During a median follow-up time of 18.0 (quartiles: 14.1, 19.3) years, 130 (10.3%) incident strokes (96 ischemic and 34 hemorrhagic) were recorded. In subclassification of the ischemic strokes, 42 (43.8%) were lacunar strokes, 27 (28.1%) were non-lacunar strokes, and 27 (28.1%) were of undetermined etiology. Of the hemorrhages, 27 (79.4%) were intracerebral hemorrhages, and 7 (20.6%) were subarachnoid hemorrhages. A total of 244 participants (19.2%) died during follow-up. The mortality was the lowest in participants with no–very mild retinopathy and the highest in participants with PDR (Table 1).

### Stroke incidence by retinopathy severity

The incidence rate for any stroke among all participants was 648 (95% CI 542–770) per 100,000 person-years. For ischemic stroke, the incident rate was 479 (95% CI 366–585) and for hemorrhagic stroke 170 (95% CI 117–237). In Kaplan-Meier analysis, the cumulative incidence of stroke by 20 years, was 5.8% in no–very mild retinopathy, 14.6% in non-proliferative retinopathy and 20.5% in PDR (*p* < 0.001). When analyzing death as a competing risk in the cumulative incidence function, the probability of stroke by 20 years of follow up was 5.5% in no–very mild retinopathy, 13.2% in non-proliferative retinopathy, and 16.7% in PDR (*p* < 0.001). The probability of death for the corresponding time was 7.5%, 16.9%, and 35.1% in no–very mild, non-proliferative retinopathy, and PDR, respectively (*p* < 0.001).

**Fig. 2:**
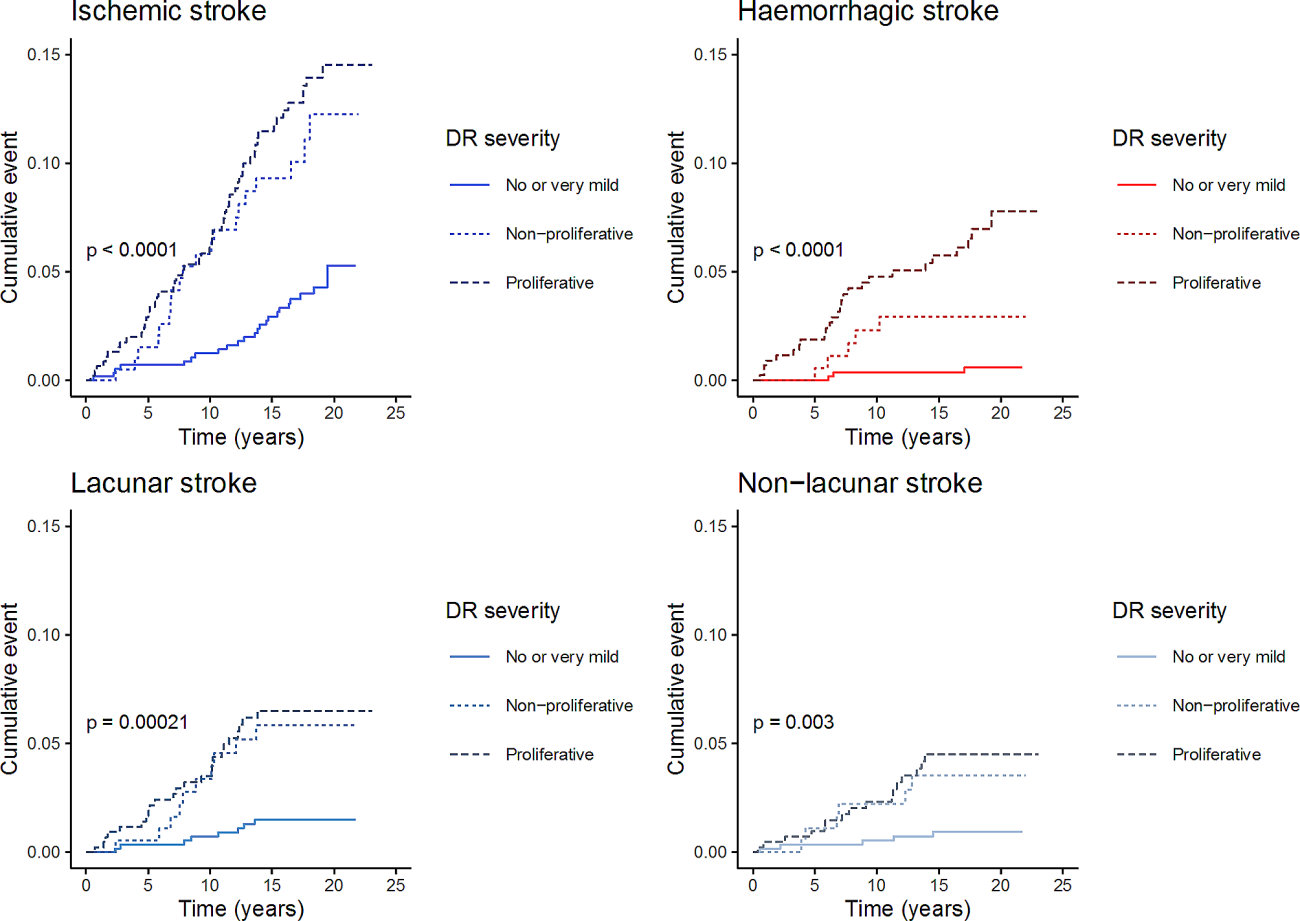
Cumulative incidence of stroke by diabetic retinopathy severity The figure presents cumulative incidence of ischemic, lacunar, non-lacunar and hemorrhagic stroke in Kaplan-Meier analyses, stratified by diabetic retinopathy severity at baseline. P represents the *p*-value of log-rank testing.

The cumulative incidence of the distinct stroke types in Kaplan-Meier analysis stratified by retinopathy severity is presented in Fig. 2. The figure displays that the proportion of events during follow-up increases by retinopathy severity (Fig. 2). In the case of hemorrhagic strokes, subtypes were not analyzed separately due to the low number of subarachnoid hemorrhages (*n* = 7).

Incidence rate and adjusted HRs for the distinct stroke types by retinopathy severity are presented in Table 2. The incidence rate for any stroke, and all types of strokes, was the highest in those with PDR at baseline, followed by participants with non-proliferative retinopathy, and the lowest in no–very mild retinopathy. The risk of any stroke was increased by 79% in non-proliferative retinopathy and by 69% in PDR, compared to no–very mild retinopathy when adjusting for sex, age, diabetes onset age, LDL cholesterol, systolic blood pressure, and presence of diabetic kidney disease. (Table 2) When analyzing the subdistribution hazarad in a Fine-Gray model with the same covariates and death as the competing event, the subdistribution HR was 1.80 (95% CI 1.02–3.18) in non-proliferative retinopathy and 1.65 (95% CI 0.971–2.79) in PDR. The subdistribution hazard for death (stroke as competing risk) was 1.72 (95% CI 1.16–2.56) in PDR and not significant in non-proliferative retinopathy (*p* = 0.230).

**Table 2:** Incidence rates and hazard ratio for stroke types by diabetic retinopathy severity

Diabetic retinopathy severity	Any stroke	Any ischemic stroke	Lacunar stroke	Non-lacunar stroke	Any hemorrhagic stroke
**No or very mild DR** **(ETDRS score < 35)**					
Incidence rate	260 (170–382)	230 (146–345)	80 (35–158)	50 (16–117)	30 (6–88)
Hazard ratio*	REF	REF	REF	REF	REF
Hazard ratio†	REF	REF	REF	REF	REF
**Non-proliferative DR** **(ETDRS score ≥ 35 and < 61)**					
Incidence rate	798 (516–1,178)	638 (390–986)	319 (153–587)	192 (70–417)	160 (52–372)
Hazard ratio*	2.16 (1.24–3.77)	2.03 (1.11–3.71)	2.77 (1.09–7.07)	2.68 (0.81–8.80)	3.54 (0.83–15.04)
Hazard ratio†	1.79 (1.02–3.15)	1.68 (0.91–3.10)	2.39 (0.93–6.14)	2.36 (0.71–7.85)	2.84 (0.66–12.28)
**Proliferative DR** **(ETDRS score ≥ 61)**					
Incidence rate	1,140 (902–1,420)	765 (573–1000)	346 (222–515)	231 (132–375)	375 (245–550)
Hazard ratio*	2.67 (1.66–4.31)	2.13 (1.26–3.60)	2.55 (1.09–5.96)	2.23 (0.78–6.33)	6.88 (1.94–24.42)
Hazard ratio†	1.69 (1.02–2.82)	1.35 (0.77–2.36)	1.72 (0.70–4.22)	1.48 (0.51–4.35)	4.31 (1.16–16.10)

Data are presented as incidence rate per 100,000 person years with (95% confidence interval). The hazard ratios (with 95% confidence interval) in the different diabetic retinopathy groups were calculated in Cox proportional hazards model.

*The analysis of any stroke was adjusted for sex, age, diabetes onset age, LDL cholesterol, and systolic blood pressure. Analyses of any ischemic lacunar, and non-lacunar strokes were adjusted for age, diabetes onset age, LDL cholesterol, and systolic blood pressure. The analysis of hemorrhagic stroke was adjusted for sex, age, diabetes onset age, triglycerides, and systolic blood pressure.

†Diabetic kidney disease added to the model

The risk of any ischemic stroke, as well as lacunar stroke, was increased both in non-proliferative retinopathy and PDR when adjusting for age, diabetes onset age, LDL cholesterol, and systolic blood pressure in a Cox model. When diabetic kidney disease was added to the model, retinopathy severity was no longer significant. Furthermore, non-lacunar stroke was not associated with any grade of diabetic retinopathy in the adjusted Cox model. (Table 2)

PDR was associated with a fourfold increase in HR for hemorrhagic stroke in comparison to no–very mild retinopathy when adjusting for sex, age, age at onset, triglycerides, systolic blood pressure, and diabetic kidney disease. Non-proliferative retinopathy did not increase the HR for hemorrhagic stroke in the model. (Table 2)

### Stroke incidence by CSME

When analyzing any stroke by presence vs. absence of CSME, the cumulative incidence was 20.8% (95% CI 15.8–25.5) in CSME and 8.8% (95% CI 5.6–14.3) in no CSME (*p* < 0.001). The HR was 1.92 (95% CI 1.31–2.82, *p* = 0.001) in CSME compared to no CSME. The analysis was adjusted for sex, age, age at onset, LDL cholesterol, and systolic blood pressure. After further adjustment for diabetic kidney disease, CSME was no longer associated with any stroke despite the nominally increased hazard (HR 1.44 [95% CI 0.97–2.15], *p* = 0.073).

**Fig. 3:**
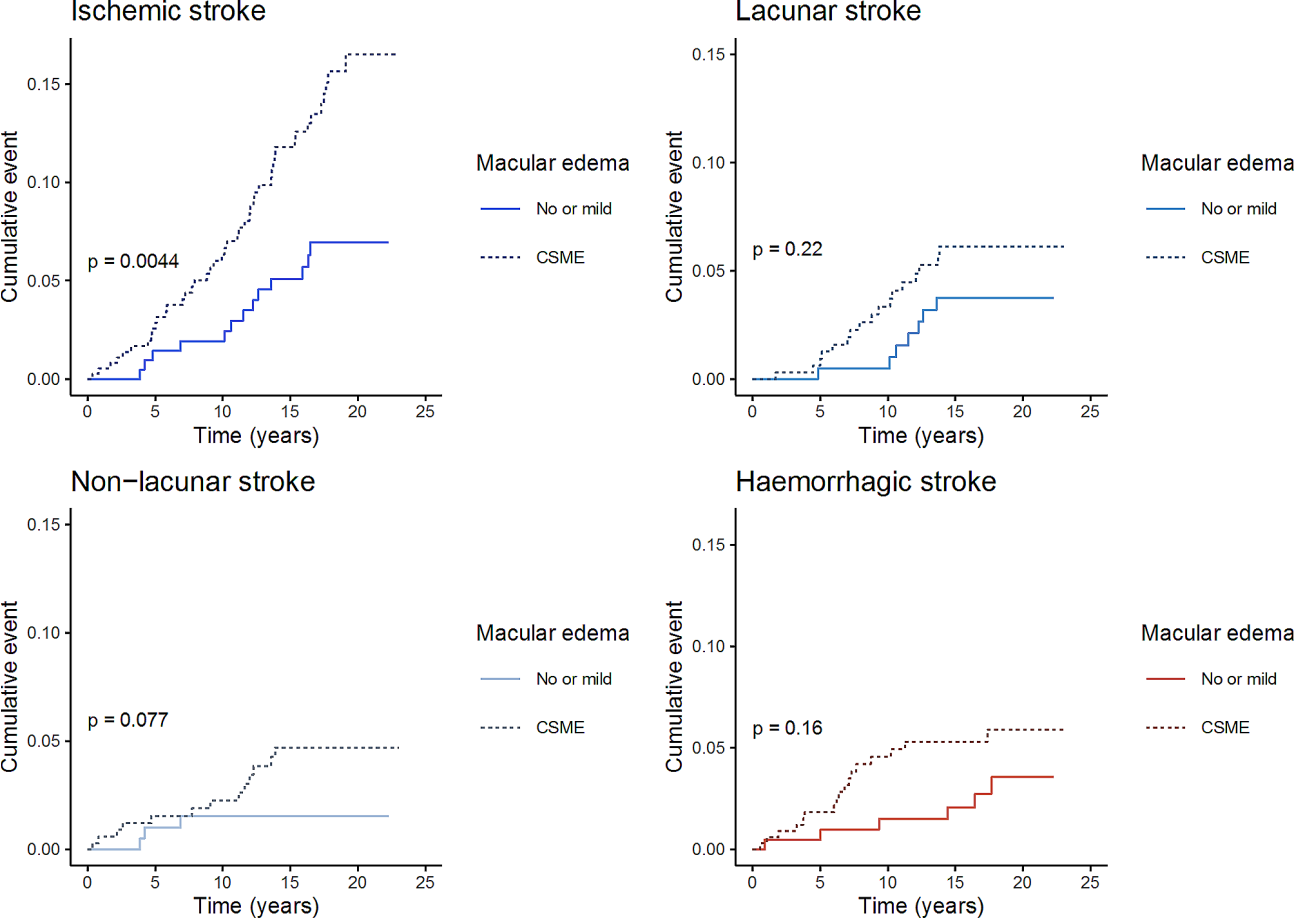
Cumulative incidence of stroke by CSME The figure presents cumulative incidence of ischemic, lacunar, non-lacunar and hemorrhagic stroke, stratified by the presence vs. absence of clinically significant macular oedema (CSME) in Kaplan-Meier analyses. P represents the *p*-value of log-rank testing.

Figure 3 presents the cumulative incidence of ischemic and hemorrhagic stroke stratified by CSME. The cumulative incidence of ischemic stroke was increased in participants with CSME compared to no CSME (Fig. 3). The HR for ischemic stroke was 1.85 (95% CI 1.19–2.88, *p* = 0.007) in CSME compared to no CSME, when adjusting for age, diabetes onset age, LDL cholesterol, and systolic blood pressure. CSME was neither associated with lacunar (*p* = 0.335) nor non-lacunar stroke (*p* = 0.140) in a similar Cox model. When adding diabetic kidney disease to the model of any ischemic stroke, CSME was no longer significant (*p* = 0.181).

CSME was associated with a higher cumulative incidence (Fig. 3) of hemorrhagic stroke the HR in CSME was 2.37 (95% CI 1.10–5.14) after adjustment for sex, age, diabetes onset age, triglycerides, and systolic blood pressure. After adding diabetic kidney disease to the model, CSME was no longer associated with hemorrhagic stroke (*p* = 0.132).

## Discussion

In our study of 1,268 adults with type 1 diabetes and ETDRS grading of diabetic retinopathy severity, we show that non-proliferative retinopathy increases the risk of stroke. Interestingly, this main observation emerged regardless of the presence of diabetic kidney disease. Furthermore, the risk associated with non-proliferative retinopathy was of the same magnitude as that of PDR. We were also able to show that the incidence of both ischemic and hemorrhagic stroke increased with the severity of diabetic retinopathy. And, as another novelty, we observed a higher incidence of stroke in participants with CSME.

Our findings are in line with previous data from the FinnDiane Study, reporting that severe diabetic retinopathy, indicated by a cruder evaluation, i.e., retinal photocoagulation, increases the risk of stroke in type 1 diabetes [13]. We now, for the first time, show that this holds true for a broader spectrum of diabetic retinopathy determined from fundus images. Furthermore, in the present study, the association was independent of diabetic kidney disease, which is somewhat surprising as diabetic kidney disease is currently one the strongest known risk factors for stroke in type 1 diabetes [3, 6].

Outside the FinnDiane Study, there is relatively limited research evaluating stroke in individuals with type 1 diabetes [2] and previous studies assessing retinopathy and stroke have shown inconclusive results [23], although retinopathy severity seems to be a risk marker for stroke in type 2 diabetes [7, 23]. The Action to Control Cardiovascular Risk in Diabetes (ACCORD) Study [7] reported that both mild and moderate to severe retinopathy are associated with stroke in type 2 diabetes and that the stroke risk increases by retinopathy severity. Their cut-off values for retinopathy severity grouping differed slightly from our study, and, furthermore, the ACCORD study did not mention diabetic kidney disease in their report. Nonetheless, we obtained similar effect sizes and, as the ACCORD Study [7], we also found the HR for stroke to increase by retinopathy severity in a Cox model not including diabetic kidney disease. In our Cox model including kidney disease, however, the HR for stroke was similar in non-proliferative retinopathy and PDR.

In our Cox model adjusting for diabetic kidney disease, however, non-proliferative retinopathy was as strongly associated with stroke as PDR. It is possible that non-proliferative retinopathy is an earlier marker of stroke in type 1 diabetes, compared to PDR, and thus might have a larger impact on the cause-specific hazard when the presence of other risk factors is low. In individuals displaying several risk factors for stroke, such as in those with PDR who also had a high prevalence of diabetic kidney disease, the impact of diabetic retinopathy might become lesser. Regardless, the significant effect of diabetic retinopathy on stroke risk was observed in both non-proliferative retinopathy and PDR in the Cox model.

In the cumulative incidence function analyzing the competing risk of stroke and death, the probability of death was higher than the probability of stroke in both non-proliferative retinopathy and PDR. Interestingly, whereas PDR was associated with death in the Fine-Gray model, non-proliferative retinopathy was not. The subdistribution hazards for stroke in the Fine-Gray model were largely unchanged compared to the Cox model, except that the association between stroke and PDR weakened slightly, so that it was non-significant, but borderline, for PDR.

We further assessed ischemic and hemorrhagic stroke, as well as subtypes of ischemic stroke, as separate outcomes in marginal analysis. For ischemic strokes, the cumulative incidence in non-proliferative retinopathy resembled that of PDR. This was particularly prominent for lacunar strokes. Moreover, in the Cox analysis not adjusted for diabetic kidney disease, the HRs in non-proliferative retinopathy and PDR were similar.

In our study, the ETDRS score indicates the mildest possible diabetic retinopathy for a participant during follow-up, as we have not assessed the progression of diabetic retinopathy. Similarities between the groups could be due to the progression of retinopathy, which the Cox model does not show. Whether or not progression was present to some degree, having non-proliferative retinopathy, i.e., more than only microaneurysms, at baseline was significantly associated with an increased risk of ischemic and lacunar stroke when diabetic kidney disease was not regarded. Non-lacunar stroke, on the other hand, was not associated with diabetic retinopathy in a corresponding model.

In the general population, lacunar strokes have been associated with retinal vascular changes in a cross-sectional setting, and it has been contemplated that they share etiological features [24]. Follow-up data on lacunar strokes and retinal microvasculature are sparse in both the general population and type 1 diabetes and although our current observations, as well as our previous report, suggest that lacunar strokes and diabetic retinopathy are linked, this relationship, particularly the hypothesized common etiology, warrants further exploration.

When analyzing hemorrhagic stroke, the cumulative incidence increased stepwise with increasing retinopathy severity. To our surprise, we did not observe a significant association between hemorrhagic stroke and non-proliferative retinopathy. This was somewhat unexpected, as we have previously reported that asymptomatic cerebral microbleeds and moderate to severe non-proliferative retinopathy are associated [12]. PDR, on the other hand, increased the HR for hemorrhagic stroke in all models in this current study, which is in line with our previous observations [6]. PDR and diabetic kidney disease might largely represent the same participants in our current cohort, in which case the impact of milder retinopathy alone may be insignificant in comparison. Considering that we have previously reported that severe diabetic retinopathy and diabetic kidney disease combined increase the risk of hemorrhagic stroke more than either complication alone [13], this might be one explanation.

The cumulative incidence of stroke was increased in participants with CSME in similarity with diabetic retinopathy. By 20 years of follow-up, the cumulative incidence had reached 21% in participants with CSME, compared to 15% in non-proliferative retinopathy and 20% in PDR. In contrast to diabetic retinopathy, CSME did not increase the risk of stroke in a Cox model adjusted for diabetic kidney disease. In analysis of ischemic stroke, the cumulative incidence at 20 years was 17% in CSME, compared to 15% in PDR, and both variables were associated with an increased HR when diabetic kidney disease was not taken into account. For hemorrhagic stroke, the cumulative incidence was 6% in participants with CSME and 8% in PDR, but in contrast to PDR, CSME was not a risk factor for hemorrhagic stroke when kidney disease was regarded.

Possibly, we were unable to detect an existing association, as macular data was available only for a subset (85%) of our participants. The prevalence of any stroke, or stroke type, did not, however, differ between those with vs. without information on macular status. Although macular edema and severe diabetic retinopathy often coincide, their risk factors also differ somewhat, e.g., whereas high systolic blood pressure increases the risk of CSME, PDR develops independent of systolic blood pressure [25]. It should therefore not be ruled out that macular edema and diabetic retinopathy represent different risk profiles. In type 2 diabetes, cardiovascular disease seems more strongly linked to macular edema than to PDR, particularly when it comes to fatal coronary heart events. Yet, the link between macular edema and stroke is unknown in type 2 diabetes as well [8]. 

In this study, we were able to thoroughly classify all incident strokes from medical records. For those without stroke, we did not confirm stroke-free status further, and some might have asymptomatic cerebrovascular disease, such as silent lacunar strokes or a history of transient ischemic attacks. We have, however, excluded all individuals for whom the clinical and radiological observations were not in agreement, the date of stroke or stroke type could not be determined, or when information regarding stroke was otherwise insufficient.

While we consider our well characterized study cohort a strength of this study, it also comes with limitations. As the study cohort is derived from a Finnish population of adults with type 1 diabetes, our results may not be generalizable to other geographical and ethnical contexts, or to other age groups.

Furthermore, although diabetic retinopathy is well characterized in our participants and grading has been done by an experienced ophthalmologist, our data are limited to information available from fundus images and medical records and do not include optical coherence tomography (OCT), for example. More detailed fenotyping based on OCT or OCT Angiography might have provided us with additional information for further, or more precise, categorization of the participants. Regardless, as screening by fundus imaging is the gold standard for detecting diabetic retinopathy to date [26], diabetic retinopathy evaluation from retinal images can still be considered highly relevant. Retinal images could, thus, serve as an accessible tool for the assessment of cerebrovascular health.

## Conclusions

In conclusion, we show that diabetic retinopathy of at least mild to severe grade, i.e., having more than only microaneurysms, increases the risk of stroke in individuals with type 1 diabetes. For the first time, we present the incidence of different stroke types, and subtypes, in relation to retinopathy severity graded by the ETDRS scale and macular edema. Our observations suggest that diabetic retinopathy, already of moderate grade, is a marker of cerebrovascular disease, and in addition to other known risk factors, may be clinically useful for the profiling of individuals at risk.

### Electronic supplementary material

Below is the link to the electronic supplementary material.

Supplementary Material 1

Supplementary Material 2

## Data Availability

Individual-level data for the study participants are not publicly available because of the restrictions due to the study consent provided by the participant at the time of data collection. The Readers may propose collaboration to research the individual level data with correspondence with the lead investigator.

## Abbreviations

CI: Confidence interval
CSME: Clinically significant macular edema
ETDRS: Early Treatment Diabetic Retinopathy Study
HR: Hazard ratio
PDR: Proliferative diabetic retinopathy
