# Supplementary material for: Stroke incidence increases with diabetic retinopathy severity and macular edema in type 1 diabetes
Source: Cardiovasc Diabetol. 2024 Apr 25;23:136. doi: 10.1186/s12933-024-02235-w (PMC11046873; doi:10.1186/s12933-024-02235-w)
Supplement: Supplementary file 1 — Supplementary Material 1 [file 12933_2024_2235_MOESM1_ESM.docx]

Supplementary Table 1

Clinical characteristics of participants included *vs* excluded from our study

|  | *n* in analysis | Included | Excluded | P |
| --- | --- | --- | --- | --- |
| n |  | 1,268 | 3,687 |  |
| Baseline data |  |  |  |  |
| Men | 4,955 | 656 (51.7) | 1,906 (51.7) | 1.000 |
| Age, years | 4,955 | 38.7 ± 10.8 | 38.0 ± 12.7 | 0.095 |
| Duration of diabetes, years | 4,955 | 25.5 ± 9.7 | 21.4 ± 13.2 | <0.001 |
| Age at onset of diabetes, years | 4,955 | 12.0 [7.3, 17.5] | 14.7 [9.6, 23.4] | <0.001 |
| Body mass index, m2/kg | 4,817 | 25.4 ± 3.6 | 25.0 ± 3.7 | 0.001 |
| HbA1c, mmol/mol | 4,787 | 71 ± 16 | 67 ± 16 | <0.001 |
| Total cholesterol, mmol/l | 4,874 | 5.1 ± 1.0 | 4.8 ± 1.0 | <0.001 |
| LDL cholesterol, mmol/l | 4,867 | 3.2 ± 0.9 | 2.9 ± 0.9 | <0.001 |
| HDL cholesterol, mmol/l | 4,870 | 1.3 ± 0.4) | 1.4 ± 0.4 | <0.001 |
| Triglycerides, mmol/l | 4,872 | 1.1 [0.8, 1.6] | 1.0 [0.8, 1.4] | <0.001 |
| Use of lipid lowering medication | 4,952 | 147 (11.6) | 566 (15.6) | 0.001 |
| Systolic blood pressure, mmHg | 4,803 | 136 ± 19 | 134 ± 19 | 0.001 |
| Diastolic blood pressure, mmHg | 4,802 | 81 ± 10 | 79 ± 10 | <0.001 |
| Use of antihypertensive medication | 4,898 | 661 (52.2) | 1304 (35.9) | <0.001 |
| Diabetic kidney disease | 4,948 | 425 (33.5) | 679 (18.5) | <0.001 |
| Coronary artery disease | 4,923 | 78 (6.2) | 244 (6.7) | 0.567 |
| Peripheral arterial disease | 4,922 | 75 (5.9) | 156 (4.3) | 0.021 |
| Follow up-data |  |  |  |  |
| Follow up time | 4,943 | 17.9 [14.0, 19.3] | 15.3 [7.6, 17.5] | <0.001 |
| Any stroke | 4,943 | 130 (10.3) | 301 (8.2) | 0.029 |
| Ischemic stroke | 4,943 | 96 (7.6) | 235 (6.4) | 0.168 |
| Hemorrhagic stroke | 4,943 | 34 (2.7) | 64 (1.7) | 0.051 |

The table presents baseline characteristics follow-up data for included participants compared to the rest of FinnDiane Study participants. Data are presented as mean ± standard deviation, median [quartiles], and n (%). Normally distributed continuous variables have been analyzed with one way ANOVA, non-normal variables with Kruskal-Wallis test and categorical data with the chi-squared test or Fisher’s exact test if observations were ≤5 in one of the groups.
