# Supplementary material for: Stroke incidence increases with diabetic retinopathy severity and macular edema in type 1 diabetes
Source: Cardiovasc Diabetol. 2024 Apr 25;23:136. doi: 10.1186/s12933-024-02235-w (PMC11046873; doi:10.1186/s12933-024-02235-w)
Supplement: Supplementary file 2 — Supplementary Material 2 [file 12933_2024_2235_MOESM2_ESM.docx]

Supplementary Table 2

Clinical characteristics presented by the status of macular edema.

|  | *n* in analysis | No macula data | P | n in analysis | no CSME | CSME | P* |
| --- | --- | --- | --- | --- | --- | --- | --- |
| n | 1268 | 194 |  | 1074 | 688 | 386 |  |
| Baseline data |  |  |  |  |  |  |  |
| Women, n (%) | 1268 | 118 (60.8) | <0.001 | 1074 | 337 (49.0) | 157 (40.7) | 0.011 |
| Age, years | 1268 | 37.0 ± 11.3 | 0.015 | 1074 | 37.16 ± 10.8 | 42.4 ± 9.7 | <0.001 |
| Duration of diabetes, years | 1268 | 24.3 ± 10.5 | 0.058 | 1074 | 24.3 ± 9.8 | 28.2 ± 8.4 | <0.001 |
| Age at onset of diabetes, years | 1268 | 12.7 ± 7.6 | 0.314 | 1074 | 12.8 ± 8.0 | 14.2 ± 8.2 | 0.009 |
| Body mass index, m2/kg | 1266 | 24.8 ± 3.2 | 0.009 | 1072 | 25.4 ± 3.8 | 25.7 ± 4.0 | 0.181 |
| HbA1c, mmol/mol (%) | 1268 | 70 ± 16  (8.6 ± 1.5) | 0.748 | 1074 | 70 ± 16  (8.6 ± 1.5) | 71 ± 16  (8.7 ± 1.5) | 0.407 |
| Total cholesterol, mmol/l | 1268 | 5.0 ± 1.0 | 0.082 | 1074 | 5.0 ± 1.0 | 5.3 ± 0.9 | <0.001 |
| LDL cholesterol, mmol/l | 1268 | 3.1 ± 0.9 | 0.014 | 1074 | 3.2 ± 0.9 | 3.4 ± 0.8 | <0.001 |
| HDL cholesterol, mmol/l | 1268 | 1.4 ± 0.4 | 0.001 | 1074 | 1.3 ± 0.4 | 1.2 ± 0.4 | 0.006 |
| Triglycerides, mmol/l | 1268 | 1.0 [0.8, 1.5] | 0.011 | 1074 | 1.1 [0.8, 1.6] | 1.3 [1.0, 1.9] | <0.001 |
| Lipid-lowering medication, n (%) | 1267 | 9 (4.6) | 0.002 | 1073 | 62 (9.0) | 76 (19.7) | <0.001 |
| Systolic blood pressure, mmHg | 1268 | 133 ± 18 | 0.022 | 1074 | 133 ± 18 | 142 ± 21 | <0.001 |
| Diastolic blood pressure, mmHg | 1266 | 80 ± 9 | 0135 | 1072 | 81 ± 10 | 82 ± 11 | 0.099 |
| Antihypertensive medication | 1267 | 73 (37.0) | <0.001 | 1074 | 295 (42.9) | 293 (76.1) | <0.001 |
| Diabetic retinopathy |  |  |  |  |  |  |  |
| ETDRS score | 1268 | 30 [15, 40] | <0.001 | 1074 | 30 [20, 62] | 65.00 [46, 75] | <0.001 |
| Any diabetic retinopathy | 1268 | 144 (74.2) | <0.001 | 1074 | 589 (85.6) | 386 (100.0) | <0.001 |
| Proliferative diabetic retinopathy | 1268 | 41 (21.1) | <0.001 | 1074 | 188 (27.3) | 261 (67.6) | <0.001 |
| Diabetic kidney disease | 1268 | 51 (26.3) | 0.007 | 1074 | 175 (25.4) | 219 (56.7) | <0.001 |
| Coronary artery disease | 1268 | 6 (3.1) | 0.066 | 1074 | 38 (5.5) | 36 (9.3) | 0.025 |
| Peripheral arterial disease | 1268 | 8 (4.1) | 0.325 | 1074 | 27 (3.9) | 40 (10.4) | <0.001 |
| Smoking status | 1246 |  | 0.009 | 1054 |  |  | 0.015 |
| Current smoker |  | 53 (27.6) |  |  | 159 (23.6) | 91 (24.0) |  |
| History of smoking |  | 31 (16.1) |  |  | 161 (23.9) | 119 (31.4) |  |
| No history of smoking |  | 108 (56.2) |  |  | 355 (52.6) | 169 (44.6) |  |
| Follow-up data |  |  |  |  |  |  |  |
| Any stroke | 1268 | 19 (7.4) | 0.920 | 1074 | 49 (7.1) | 62 (16.1) | <0.001 |
| Ischemic stroke | 1268 | 13 (6.7) | 0.726 | 1074 | 38 (5.5) | 45 (11.7) | <0.001 |
| Type of ischemic stroke | 96 |  | 0.802 | 83 |  |  | 0.573 |
| Lacunar stroke |  | 7 (53.8) |  |  | 18 (47.4) | 17 (37.8) |  |
| Non-lacunar stroke |  | 3 (23.1) |  |  | 11 (28.9) | 13 (28.9) |  |
| Undefined ischemic stroke |  | 3 (23.1) |  |  | 9 (23.7) | 15 (33.3) |  |
| Hemorrhagic stroke | 1268 | 6 (3.1) | 0.886 |  | 11 (1.6) | 17 (4.4) | 0.010 |
| Type of hemorrhagic stroke | 33 |  | >0.999 | 28 |  |  | 0.653 |
| Intracerebral hemorrhage |  | 5 (2.6) |  |  | 8 (72.7) | 14 (82.4) |  |
| Subarachnoid hemorrhage |  | 1 (0.5) |  |  | 3 (27.3) | 3 (17.6) |  |
| Death during follow-up |  | 30 (15.5) | 0.176 | 1074 | 89 (12.9) | 125 (32.4) | <0.001 |

Supplementary Table 2 presents baseline and follow-up characteristics grouped by macular status: No macula data; no clinically significant macular edema (CSME); and CSME. P indicates the p-value from tests comparing participants with vs without macular data. For participants with macula data available, the characteristics are presented according to presence vs absence of CSME. P* indicates the p-value in tests comparing participants with *vs* without CSME. Data are presented as mean ± standard deviation, median [quartiles] and number (percent). Normally distributed continuous variables have been analyzed with one way ANOVA, non-normal variables with Kruskal-Wallis test and categorical data with the chi-squared test or Fisher’s exact test if observations were ≤5 in one of the groups.
